# Absent Ductus Venosus Associated with Partial Liver Defect

**DOI:** 10.1155/2018/6591025

**Published:** 2018-06-13

**Authors:** Kenji Horie, Hironori Takahashi, Daisuke Matsubara, Koichi Kataoka, Rieko Furukawa, Yosuke Baba, Akihide Ohkuchi, Shigeki Matsubara

**Affiliations:** ^1^Department of Obstetrics and Gynecology, Jichi Medical University, Japan; ^2^Department of Pediatrics, Jichi Medical University, Japan; ^3^Department of Radiology, Jichi Medical University, Japan

## Abstract

Absent ductus venosus (ADV) is a rare vascular anomaly. We describe a fetus/neonate with ADV with a partial liver defect. A 41-year-old woman was referred to our institute because of fetal cardiomegaly detected by routine prenatal ultrasound, which revealed absence of ductus venosus with an umbilical vein directly draining into the right atrium, consistent with extrahepatic drainage type of ADV. She vaginally gave birth to a 3,096-gram male infant at 38 weeks of gestation. Detailed ultrasound examination revealed a defect of the hepatic rectangular leaf at half a month postnatally. He showed normal development at 1.5 years of age with the liver abnormality and a Morgagni hernia. Liver morphological abnormality should also be considered as a complication of ADV.

## 1. Introduction

Absent ductus venous (ADV) occurs in 1/2,500 singleton pregnancies at a median gestation of 12^+5^ (range 11^+0^ to 13^+6^) weeks [1]. This condition frequently accompanies various congenital abnormalities including cardiac anomaly, which may adversely influence the prognosis [2]. In ADV, the umbilical vein (UV) drains to the fetal systemic venous circulation through an intra- or extrahepatic route. This abnormal drainage often causes cardiac volume overload, leading to heart failure. The drainage type (intra- or extrahepatic) was reported to affect the prognosis of fetuses/infants with ADV [3].

Here, we describe a fetus/neonate prenatally diagnosed with the extrahepatic drainage type of ADV with a partial liver defect. This is, to our knowledge, the first report on ADV with this congenital abnormality.

## 2. Case Report

**Figure 1:**
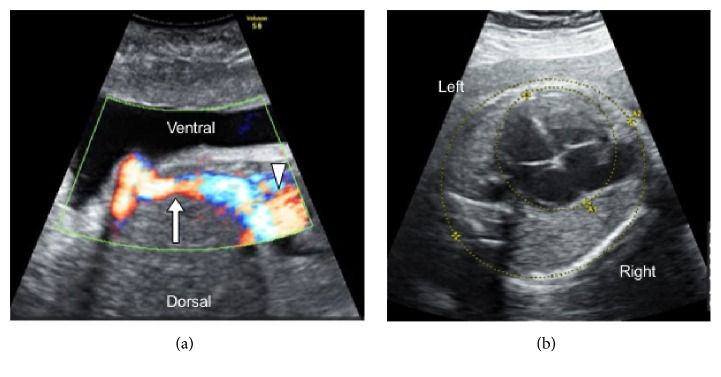
Prenatal ultrasound findings of a patient with absent ductus venosus. (a) The umbilical vein (arrow) directly flows into the right atrium (arrowhead). (b) The cardiothoracic area ratio was 36.5%.

At the 30th gestational week, a 41-year-old (gravida 2, para 1 [normal vaginal delivery]) woman with no remarkable medical/family histories was referred to us because of fetal cardiomegaly detected on routine prenatal ultrasound. Fetal ultrasound revealed the absence of ductus venosus (DV) with the UV directly draining into the right atrium (Figure 1(a)), consistent with the extrahepatic drainage type of ADV. The cardiothoracic area ratio was 36.5%, within the normal range of <40% (Figure 1(b)) and heart valve regurgitation was absent. No cardiac structural abnormalities were detected, and cardiac functional parameters were normal. The parents did not desire fetal karyotyping, and, thus, amniocentesis was not performed. Direct UV flow into the systemic venous circulation (the right atrium) usually causes volume overload of the right heart, and thereby right heart failure, whose signs were carefully monitored, but they were not observed.

**Figure 2:**
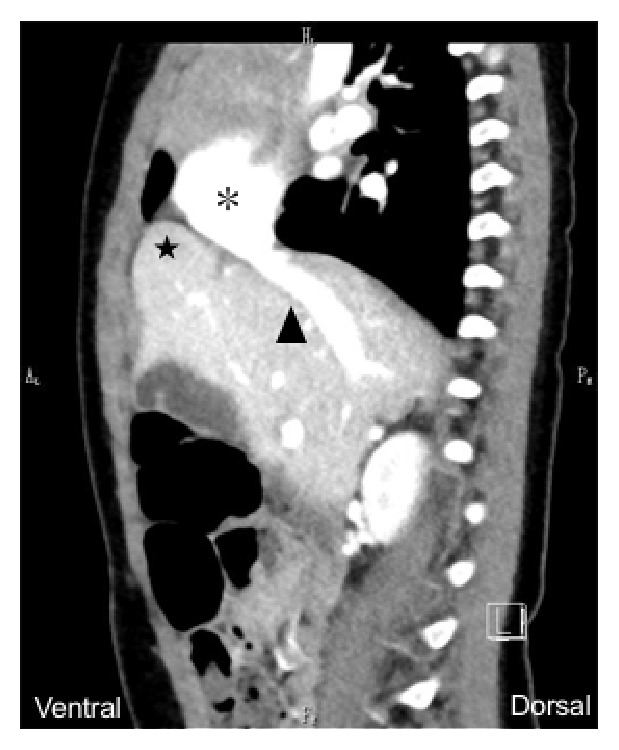
Abdominal computed tomography at 1 year of age. A Morgagni hernia (black star) can be seen, and the right atrium (asterisk) and inferior vena cava (arrowhead) are displaced on the ventral side.

At 38^+3^ weeks, she showed the spontaneous onset of labor and vaginally gave birth to a 3,096-gram male infant (Apgar score 7/8 [1/5 min]). Neonatal cardiac ultrasound revealed mild aortic valve regurgitation and a slightly decreased ejection fraction, which were transient and disappeared on day 7. Detailed ultrasound examination revealed a defect of the hepatic rectangular leaf (S4: one of the largest liver leaves) at half a month postnatally. No findings indicative of liver dysfunction were observed throughout his course. Computed tomography at 1 year of age revealed atypical liver rotation with a Morgagni hernia in the liver (Figure 2). He showed normal development at 1.5 years of age.

## 3. Discussion

This is, to our knowledge, the first report of a patient with ADV accompanied by a partial liver defect. Detailed imaging analyses revealed this rare liver abnormality.

ADV is often complicated by various congenital abnormalities, associated or unassociated with chromosomal abnormalities. Congenital cardiac abnormalities are the most common, including ventricular septal defect, valve abnormalities, double outlet right ventricle, or coarctation of the aorta [2]. Various chromosomal abnormalities are also frequently observed in ADV. ADV without congenital/chromosomal abnormalities, namely, “isolated” ADV, accounts for 35-59% of all ADV [2–4]. Isolated ADV, compared with that complicated by congenital/chromosomal abnormalities, is usually associated with a better prognosis [4]. Since cardiac or chromosomal abnormality is a predominant complication and may be the main determinant of the prognosis, physicians may focus their attention on them, being less suspicious of other organ abnormalities. Here, we demonstrated that liver abnormality can coexist with ADV.

**Figure 3:**
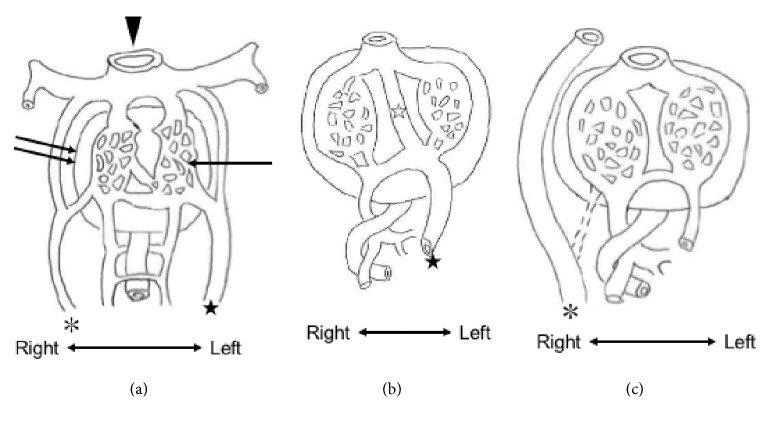
Proposed model of development in a patient with absent ductus venosus. (a) Normal fetal abdominal development at 5 weeks of gestation. Situs (arrowhead), hepatoblast (double arrows), liver sinusoid (arrow), left umbilical vein (black star), and right umbilical vein (asterisk) were indicated. (b) Normal development at 8 weeks of gestation. The right umbilical vein had regressed. Ductus venosus (DV) (white star) formed following development of the left umbilical vein (black star). (c) Abdominal development in a patient with extrahepatic absent ductus venosus at 8 weeks of gestation. The right umbilical vein (asterisk) persists and, thus, the flow of the left umbilical vein has decreased. Consequently, DV has not formed.

We believe that the partial liver defect is not a mere coincidence in the presence of ADV. This is because the liver and DV development are closely related. In the early developmental stage, there were two UVs, the right and left UVs, which run on the right and left sides of the liver (Figure 3(a)), with the right one disappearing during normal fetal development, and the left one eventually becoming DV, conveying oxygenated blood to the liver and inferior vena cava (Figure 3(b)). If there is some abnormality in this development/regression of the two-umbilical vein system, ADV occurs, in which oxygenated blood supply to the liver may decrease (Figure 3(c)). This insufficient blood supply to the liver (liver hypooxygenation) may cause malfunction/malsecretion of various differentiation-related substances including growth factors, cytokines, and proteins (e.g., Foxa1) [5]. As such, ADV may be closely related to liver development, and a partial liver defect may occur in association with ADV. Thus, we believe that the present liver abnormality may not be a coincidence but may be associated with ADV.

Persistent right UV and possibly its associated ADV with the partial liver defect may well explain the present patient's pathophysiology/etiology. However, patients with the persistence of right UV frequently have normal DV and some researchers considered this as statuses of normal anatomical variants [6]. A recent study revealed that 75% (9/12) of patients with the persistence of right UV were uneventful during delivery and postpartum period [7]. Thus, of patients with persistent right UV, some show normal DV without abnormalities (including liver abnormality) whereas others (the present case) show ADV and liver abnormalities. At present, we do not know the reason for this discrepancy. Possibly, various developmental factors (the time of derangement of normal regression of two UV system, its acuteness/abruptness, and its degree) may be associated with this. Further studies are necessary to clarify this issue.

Putting aside this discussion, previous reports emphasized cardiac anomaly and little attention may have been paid to liver anomaly, there may have been some liver defects associated with ADV, but these remained unrecognized and unreported [2, 3]. Our patient also had a Morgagni hernia. This is an anterior right-sided defect of the diaphragm. A recent report described a Morgagni hernia concomitantly seen in a patient with ADV [8]. Considering the position of the hernia (anterior, right-sided), the hernia also may be associated with ADV.

Concerning its drainage route, ADV is divided into 2 types: intra- and extrahepatic drainage types, with the latter being in the present case. In the former intrahepatic type, the UV flow enters the liver, whereas in the latter extrahepatic type, it bypasses the liver and directly enters the inferior vena cava or right atrium. Extrahepatic ADV was correlated with a significantly poorer prognosis than intrahepatic ADV [3], which was also demonstrated by a recent review based on the largest study population to date [2]: nonsurvivors were more frequent in those with extra- than intrahepatic ADV. In extrahepatic ADV, large-volume flow directly enters the systemic circulation (the inferior vena cava or right atrium), which may lead to cardiac volume overload, causing heart failure [3, 9, 10]. Some part or all of the portal system will develop in a case with ADV. Its neighboring structure may vary with the type of ADV. The extrahepatic drainage may be more closely related to the liver defect.

We report a patient with the extrahepatic drainage type of ADV with a partial liver defect. Although it remains unclear whether this liver abnormality will affect this patient in later life, physicians should perform periodic ultrasound examination of patients with ADV. Liver morphological abnormality should also be considered as a complication of ADV, although whether its prenatal/postnatal diagnosis may affect the treatment strategy is unclear. Data accumulation is needed to determine the clinical significance of this liver abnormality associated with ADV.
